# Optimizing the Water Ecological Environment of Mining Cities in the Yangtze River Economic Belt Using the Cloud Model, CV-TOPSIS, and Coupling Coordination Degree

**DOI:** 10.3390/ijerph19042469

**Published:** 2022-02-21

**Authors:** Ran Wang, Hao Lin, Jinhua Cheng, Zixi Xu, Haoying Feng, Yameng Tang

**Affiliations:** 1 School of Economic & Management, China University of Geosciences, Wuhan 430074, China; wangran2cug@163.com; 2 Research Center of Resource and Environmental Economics, China University of Geosciences, Wuhan 430074, China; linhao2020@126.com (H.L.); xuzixi163@163.com (Z.X.); haoyingfeng@cug.edu.cn (H.F.); tangyameng0413@163.com (Y.T.)

**Keywords:** Yangtze river economic belt, mining city, water ecological environment, cloud model, CV-TOPSIS, coupling coordination degree model

## Abstract

The Yangtze River Economic Belt (YREB) is the core region for the security of mineral resources in China and is a strategic water source containing rich water resources. Coordinating the security of mineral resources and water resources in the YREB is a key problem. Establishing and optimizing the water ecological environment (WEE) is crucial for addressing this problem in mining cities, which are the main bases for the supply of mineral resources. This study applies the cloud model, CV-TOPSIS, the standard deviation ellipse, and the coupling coordination degree model to evaluate the WEE and the coordinated development state, and to optimize the WEE. The results show that: (1) the WEE of mining cities in the YREB is generally good; (2) the protection of WEE in most mining cities has achieved significant results recently, and the results in the downstream are more remarkable than those in the mid-upstream; (3) the coordinated development of WEE in regenerative mining cities is better than that of mature and declining cities; and (4) most mining cities still belong to the lagging type of water environment (heavy metal pollution has been better treated and the threat of water ecological security caused by heavy metal pollution is low). This study suggests improvements to the sewer system, promotes WEE management in the mid-upstream, and propels the transformational development of mature and declining mining cities in advance.

## 1. Introduction

The Yangtze River Economic Belt (YREB) has become a major national strategic development area in China. The YREB accounts for more than 40% of the population and gross domestic product (GDP), and approximately 40% of total imports and exports in China. The YREB, which integrates mountains, rivers, forests, farmland, and lakes, has a prominent position in the ecological environment, and is an important ecological treasure-house in China. Additionally, it is a strategic and extremely rich water resource, and an important ecological safety barrier area, with soil and water conservation, and flood regulation and storage.

The report of the 19th Party Congress proposed a coordinated regional development strategy for “grasping great protection and not engaging in great development” to promote the development of the YREB [1]. The ecological environment of the YREB can only be optimized, and cannot be allowed to deteriorate. Water ecological environment (WEE) protection has become an overarching strategic issue with regard to the sustainable development of the YREB. However, due to the influence of the traditional economic development path [2], WEE in the YREB remains severe [3].

**Figure 1:**
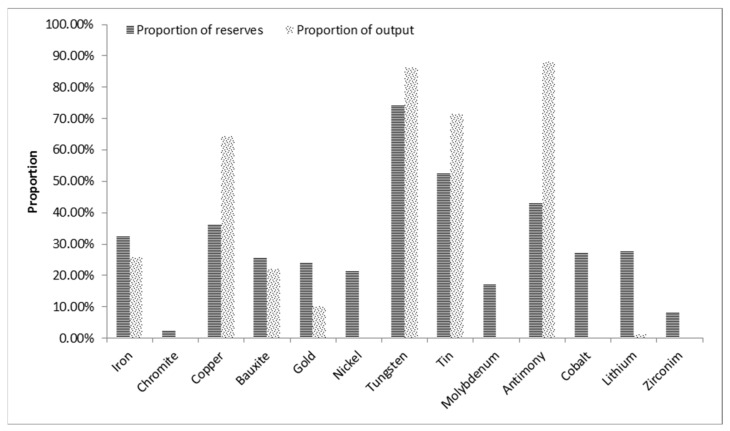
Proportion of reserves and output of strategic metal minerals in the YREB in 2016.

Meanwhile, the YREB is rich in mineral resources, and the exploitation and utilization of these resources are of great significance to the security of China’s mineral resources. Copper, tungsten, tin, and antimony account for 65.9%, 86.15%, 71.45%, and 88.06%, respectively, of the strategic production of metallic ore in the YREB in 2016. Compared with the reserve ratio, their mining intensity is relatively high, especially antimony; the mining intensity of iron, aluminum, and gold is moderate (Figure 1).

There are 98 mining cities in the YREB, which are faced with prominent environmental problems [4]. The quality of the aquatic environment of mining cities in the YREB is related to their own healthy economic and social development, and has a vital impact on the entire Yangtze River basin and the country. Mining cities should take the lead in “grasping great protection and not engaging in great development”. Therefore, evaluating and optimizing the WEE of mining cities in the YREB is crucial to achieve high-quality development. 

The contribution of this study is mainly reflected in three aspects. First, an index system for water ecological environment is constructed, which conforms to the characteristics of mining cities in the YREB. Second, CV-TOPSIS, cloud model, a standard deviation ellipse model, and a coupling coordination model are used to evaluate and optimize WEE in mining cities in the YREB. Third, the diverse characteristics of WEE in mining cities are analyzed from different timescales, spaces, and stages. The findings of this study are important for the protection of WEE and economic development of mining cities in the YREB, while providing an informative reference for policy makers.

## 2. Literature Review

Existing related research mainly focuses on evaluating the water environment and the ecological environment quality. Research on the water environment quality is relatively mature, including the evaluation of key indices and a comprehensive index [5,6]. The water environment comprises spaces surrounding people or surrounding water bodies, and it can directly or indirectly affect human life and development [7]. Dos et al. (2018) selected the concentration of pollutants, such as chemical oxygen demand (COD) and ammonia nitrogen (NH_4_-N), as the key indices for evaluating water environmental quality [8]. Avila et al. (2018) regarded the quantity of *Escherichia coli* as an indicator of fresh water quality, and believed that the growth in the quantity of *Escherichia coli* increases the risk of disease [9]. Li et al. (2011) explored the water environment quality using the proportion of water quality sections in the basin, such as the proportion of water above class III (inclusive), and inferior class V water quality [10]. The calculation of a composite index based on the construction of an index system has also been applied in many studies [11,12,13], and pollutants, such as COD, NH4-N, DO, and TN, have been considered in the literature [14,15,16].

With the introduction of the “five-in-one” strategy for ecological civilization, environmental protection has been raised to unprecedented heights. Recently, studies on ecological and environmental protection have increased significantly [17,18], including studies related to WEE protection. Water ecosystems support a wide range of organisms, including bacteria, fungi, algae, plants, invertebrates, and fish [19]. Zuo et al. (2021) selected total water resources per capita, waste water discharge per 10,000 yuan of GDP, sewage treatment rate, and other indicators for constructing China’s provincial ecological civilization evaluation index system [20]. Liu et al. (2018) selected total water resources per capita, waste water discharge, and other indicators for building an ecological environment assessment system [21]. Li et al. (2021) selected industrial wastewater emissions per CNY 100 million GDP, industrial SO_2_ emissions per CNY 100 million GDP, and smoke and dust emissions per CNY 100 million GDP as the indicators for environmental evaluation [22].

The development of mining cities in the YREB has attracted attention in many studies [23,24,25,26,27], including sustainable development of the WEE. Li et al. (2016) studied the distribution characteristics of heavy metal pollution in a copper mining area of Yalong River through field sampling surveys, combing with GIS and remote sensing technology [28]. Zhang et al. (2011) and Dong et al. (2014) regarded the tributaries of the Yangtze River as the research subjects for evaluating heavy metal pollution [29,30]. Chen et al. (2021) considered Huainan city, which is one of the mining cities in YREB, as the research subject for evaluating the genesis and dominant processes of groundwater salinization [31].

The coupling coordination degree model has been used in various fields of ecological civilization construction [32,33,34,35]. Additionally, some scholars have chosen one aspect of the system to study. Li et al. (2021) and Yang et al. (2020) assessed the coupling and coordinated development state of the rural production–living–ecological function by considering the territorial space system as the research subject [10,36]. Zuo et al. (2021) evaluated the coordinated development state of the economy, society, and nature in the ecological civilization system [20]. Dong and Li (2021) evaluated the coupling coordination degree of “up-mid-downstream” of China’s wind power industry chain [37]. Therefore, this study analyzes the coordinated development of the WEE system of mining cities in the YREB by referring to the coupling coordination degree model. Additionally, the TOPSIS model is used.

The TOPSIS model selects a limited number of evaluation indicators based on the characteristics of the evaluation object. Subsequently, it chooses the ideal value for each indicator before calculating the distance between each solution and the ideal value. This allows it to determine the best solution by considering the strengths and weaknesses of each evaluation object [38,39,40]. Similar to TOPSIS, stable preference ordering towards ideal solutions (SPOTISs) and the characteristic object method (COMET) use reference objects to determine preferences for any alternatives. They mainly focus on being resistant to the rank reversal phenomenon [41,42]. The main advantage of the TOPSIS algorithm over other methods is that it makes full use of raw data. Additionally, its results accurately reflect the gaps between the various assessment options. Furthermore, the simpler data handling and ease of computational processing of TOPSIS makes it widely applicable [43,44,45]. Consequently, the model has been used extensively used in assessing river health [46,47,48].

Throughout the existing literature, the evaluation of the water environment has increasingly attracted the attention of scholars, and some research focusing on WEE protection of the provinces and cities in the YREB exists. However, the focus on protecting WEE of mining cities in the YREB is insufficient, especially the different characteristics of WEEs. Based on this, the main innovation points of this study include: (1) constructing a unique index system for evaluating WEEs for mining cities in the YREB; (2) analyzing the diverse characteristics of WEEs of mining cities in diverse times, spaces, and stages; and (3) analyzing the coordinated development state in WEE systems.

## 3. Study Area and Method

### 3.1. Study Area

**Figure 2:**
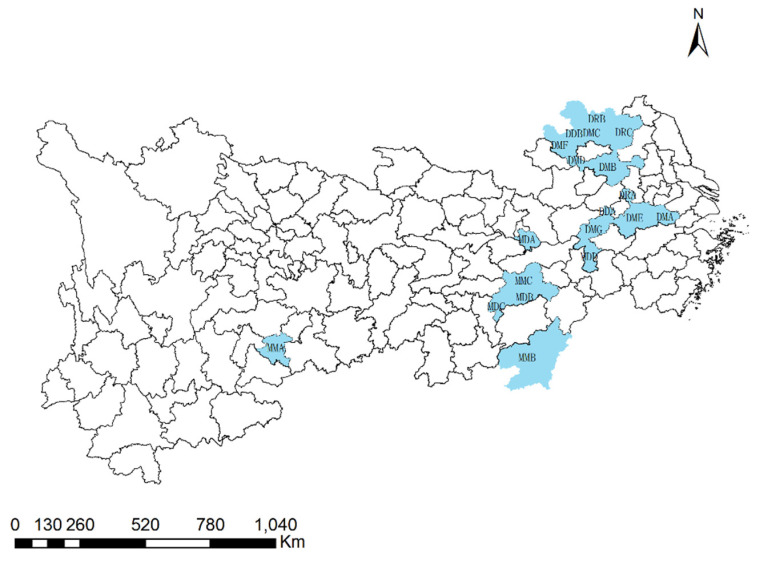
Study areas.

Mining cities are those established near to or within regions rich in mineral resources, and have ore mining, processing, and/or smelting as the main industry [49]. Based on the ideas of distribution pattern, occurrence characteristics, and the development stage, combined with the principle of data accessibility, 19 mining cities were selected as the study areas (Figure 2). The names, regions, development stages, and codes of each mining city are shown in Table 1. The first letter, M or D, denotes mid-upstream or downstream, the second letter, M, D, or R, denotes mature, declining and regenerative, and the third letter (for example, A, B, and C) denotes the ordinal.

The specific selection criteria were based on the following ideas: (1) prefecture-level cities were considered for the site selection. There are 44 mining cities at prefecture level in YREB, and about 50% (19) of the total mining cities were selected based on the accessibility of data. (2) Focus was placed on non-growing mining cities that were not growing with reference to their development stages. Growing mining cities are in the rising stage of resource development, and the impacts of mineral resource development on the WEE have not been highlighted. In contrast, mature, declining, and regenerating mining cities face greater problems of ecological environment and economic transformation. Hence, the present study mainly focused on such mature mining cities.

### 3.2. Research Framework

**Figure 3:**
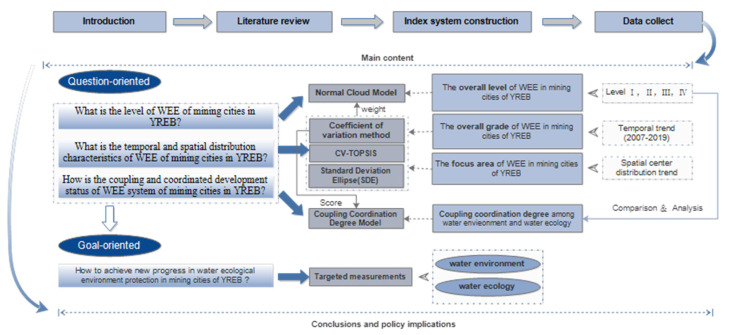
Research framework.

For achieving a better WEE of mining cities in the YREB, this study attempted to answer the following three questions: (1) what is the level of WEE systems of mining cities in the YREB? (2) What are the temporal and spatial distribution characteristics of WEE systems of mining cities in the YREB? (3) What is the coordinated development state of WEE systems of mining cities in the YREB? Therefore, based on the problem-oriented background, the cloud model, coefficient of variation (CV)-TOPSIS, the standard deviational ellipse (SDE) model, and the coupling coordination degree model are used to answer these questions. The research framework is shown in Figure 3.

### 3.3. Construction of an Index System

In April 2018, the Chinese President convened a symposium to promote the development of the YREB, stressing the need to solidly promote the control of water pollution, the restoration of water ecology, and the protection of water resources. In 2020, academician Wang Jinnan proposed “five-water integration” of water resources, water environment, water ecology, water disasters, and water regulation. Whether it is the “three waters” or “five waters”, the top priorities are given to water resources, water environment, and water ecology. The YREB is very rich in water resources and, hence, the present study focuses on the WEE protection, which includes both water environment and water ecology. Water environment focuses on indicators of discharge of a water pollutant and its management, and water ecology focuses on indicators that are more likely to have a negative impact on aquatic plants and animals.

The wastewater discharge of various mines in YREB has a great impact on WEEs in the river basin. Our research group summarized the impact factors of mineral resources development on the WEEs of each province and city in the YREB, based on the investigation. It can be observed that surface water and groundwater pollution in mining cities is relatively serious, especially the latter (Table 2). Water sources and reservoirs are polluted to varying degrees, and aquatic life and human settlements are facing great challenges. There were mining agglomerations in mining cities that discharged the sewage directly into the Yangtze River. For example, Dongchuan district, which is located in the mining city of Yunnan Province, connected a sewage pipe to the Yangtze River; Huidong County of Liangshan Yi Autonomous Prefecture, which is in the mining city of Sichuan Province, fed the wastewater of mineral separation into the tail water of a power plant and dumped it into the Jinsha River. These behaviors polluted the water body and threatened the water environment of the Yangtze River.

**Figure 4:**
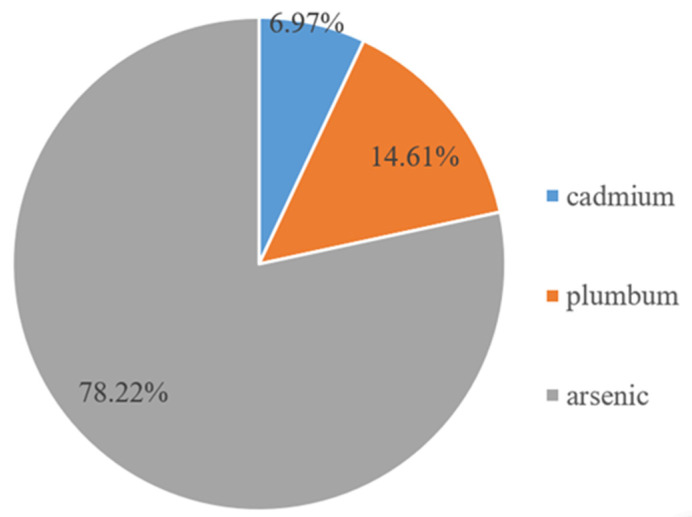
Proportion of heavy metal pollution in the YREB.

Water pollution in mining cities has a strong negative impact on the quantity and quality of aquatic animals and plants [50,51]. The group incidents and safety problems of agricultural products, such as cadmium rice and heavy metal vegetables, caused by heavy metal pollution are increasing annually, threatening the safety of aquatic organisms and anthropogenic settlements. For example, cadmium rice appeared in Hunan, Jiangxi, Guangdong, and Guangxi, and there were 161 batches of unqualified food in Guangxi. In 2016, the heavy metal emissions from non-ferrous metal mining and dressing in the YREB totaled 323.87 kg, among which cadmium, plumbum, and arsenic amounted to 22.27 kg, 47.32 kg, and 253.36 kg, respectively, accounting for 6.97%, 14.61%, and 78.22% of the heavy metal emissions, respectively (Figure 4).

The construction of the index system is the core of WEE quality evaluation [52]. Based on the above considerations and combined with the principles of scientific, operability, and authoritativeness of data sources, the index system for evaluating the WEE of mining cities in the YREB can be constructed from two aspects: water environment and ecology. Given the impact of industrial development in mining cities, indices of water environment dimension are selected from the discharge of industrial wastewater, industrial sulfur dioxide, industrial (tobacco) dust, industrial COD, and ammonia nitrogen. China is promoting new infrastructures, and the length of sewage pipelines is considered as a part of the infrastructure for water environmental management. Given the particularity of mineral resources development and utilization in mining cities, the emphasis of the water ecology dimension is placed on the heavy metal pollution that affects the safety of aquatic organisms and humans; thus, the indicators of discharge of mercury, cadmium, plumbum, and arsenic were selected. The indicators and their attributes are shown in Table 3.

### 3.4. Cloud Model

The cloud model is a decision-making method that can establish the transformation between qualitative and quantitative concepts. Additionally, it can reflect the randomness and fuzziness of comprehensive evaluation [53]. The cloud model has been previously used for risk assessments [54] and disaster assessments [55]. Considering the characteristics of the evaluation of WEE systems, the cloud model can be applied. This method reflects the qualitative concept quantitatively by cloud digital features (Ex, En, He). Ex is the expected value of the evaluation results; En is entropy reflecting the fuzzy degree of the evaluation results; and He is hyper-entropy reflecting the discreteness of entropy. This method uses the reverse cloud generator to convert accurate values into cloud model parameters and uses the forward cloud generator to convert cloud parameters into cloud droplets, and finally forms a cloud chart [56]. The following steps should be used to draw the cloud chart using Python software.

Build the standard cloud:

(1) $$\{\begin{array}{c} Ex_{0}=\frac{Q_{min}+Q_{max}}{2}\\ En_{0}=\frac{Q_{max}-Q_{min}}{2}\\ He_{0}=b \end{array},$$

(2) $$Ex_{j}^{o}=\frac{\sqrt{\sum_{j=1}^{n}{\left(U_{ij}-U_{j}^{o}\right)}^{2}}}{\sqrt{\sum_{j=1}^{n}{\left(U_{ij}-U_{j}^{o}\right)}^{2}}+\sqrt{\sum_{j=1}^{n}{\left(U_{ij}-U_{j}^{*}\right)}^{2}}},\;$$

*Q_max_*, *Q_min_* represent the upper and lower limits of the evaluation interval, respectively; ($U_{j}^{o},\;Ex_{j}^{o}$) represent the cloud droplets; and b is a constant that represents the standard value of super entropy. The value of b is 0.001 [57].

Calculate the indicator cloud parameters:

(3) $$\{\begin{array}{c} {\bar{X}}_{j}=\frac{1}{k}\overset{k}{\underset{i=1}{\sum }}x_{ij}\\ S_{j}^{2}=\frac{1}{k-1}\overset{k}{\underset{i=1}{\sum }}{\left(x_{ij}-\bar{X}\right)}^{2}\\ Ex_{j}={\bar{X}}_{j}\\ En_{j}=\sqrt{\frac{\pi }{2}}\cdot \frac{1}{k}\overset{k}{\underset{i=1}{\sum }}\left|x_{ij}-{\bar{X}}_{j}\right|\\ He_{j}=\sqrt{\left|S_{j}^{2}-En_{j}^{2}\right|} \end{array},$$

*X_ij_* is sample data; ${\bar{X}}_{j}$ is sample mean; $S_{j}^{2}$ is the sample variance; $Ex_{j}$ is the expected value; $En_{j}$ is entropy; and $He_{j}$ is hyper-entropy.

Calculate the comprehensive cloud parameters:

(4) $$\{\begin{array}{c} Ex=\overset{n}{\underset{j=1}{\sum }}Ex_{j}z_{j}\\ En=\sqrt{\overset{n}{\underset{j=1}{\sum }}En_{j}^{2}z_{j}}\\ He=\overset{n}{\underset{j=1}{\sum }}He_{j}z_{j} \end{array},$$

where *Z_j_* represents the weight of the index combination obtained by the coefficient of variation (CV) method. *Ex* is the expected value of the comprehensive cloud parameters; *En* is entropy; and *He* is hyper-entropy.

Cloud models are relatively new tools for studying uncertain knowledge and converting qualitative concepts and quantitative dates [58]. As the fuzzy cloud model evaluations can effectively evaluate WEE systems, the present study applied the method to the evaluation of WEE systems, and the specific classification standard is shown in Table 4.

The three numbers in the standard cloud parameters are (*Ex*, *En*, *He*) where *Ex* is the expected value of the evaluation results; *En* is the entropy reflects the fuzzy degree of the evaluation results; and *He* is the hyper-entropy that reflects the discreteness of entropy. The value of *He* is 0.001 refers to the reference. For example, for level 1, 0.125 is the expected value, 0.0736 is the entropy, and 0.001 is the hyper-entropy.

### 3.5. Standard Deviational Ellipse Model

The SDE model analyzes the distribution characteristics of discrete point data using a rotated ellipse with a long axis, to denote the main orientation of a discrete data [59]. SDE can elucidate the center of gravity shift tendencies in an area using a standard planar coordinate system (*X*, *Y*) with any discrete point having the coordinates (*x_i_*, *y_i_*). The long half axis of the ellipse represents the direction of data distribution, and the short half axis represents the range of data distribution. The larger the value difference between the long and short half axes, the more obvious the data directionality. In contrast, the closer the long and short half axes are, the less obvious the directivity. The short half axis indicates the range of data distribution. The shorter the short half axis, the more obvious the centripetal force is. In contrast, the longer the short semi axis, the greater the degree of dispersion of the data. The SDE model has been previously used for ecological environment assessment [60] and environmental management [61].

The SDE model reflects the spatial distribution characteristics of WEE quality from multiple angles. SDE was selected for this study because it reveals changes in the spatial distribution range of WEE quality through the comparative areas of the standard elliptical difference. The calculation formula is as follows:

Ellipse center coordinates:

(5) $$SDE_{x}=\sqrt{\frac{\sum_{i=1}^{n}{\left(x_{i}-\bar{X}\right)}^{2}}{n}},\;$$

(6) $$SDE_{y}=\sqrt{\frac{\sum_{i=1}^{n}{\left(y_{i}-\bar{Y}\right)}^{2}}{n}},\;$$

*x_i_* and *y_i_* are the spatial position coordinate of each feature, and *X* and *Y* are the arithmetic mean center.

Direction of ellipse:

(7) $$\tan\theta =\frac{A+B}{C},$$

(8) $$A=\sum_{i=1}^{n}{\tilde{x}}_{i}^{2}-\sum_{i=1}^{n}{\tilde{y}}_{i}^{2},$$

(9) $$B=\sqrt{{\left(\sum_{i=1}^{n}{\tilde{x}}_{i}^{2}-\sum_{i=1}^{n}{\tilde{y}}_{i}^{2}\right)}^{2}}+4{\left(\sum_{i=1}^{n}\tilde{x_{i}}\tilde{y_{i}}\right)}^{2},\;$$

(10) $$C=2\sum_{i=1}^{n}\tilde{x_{i}}\tilde{y_{i}},\;$$

where *θ* represents the azimuth of the ellipse, $\tilde{x_{i}}$ and $\tilde{y_{i}}$ are the difference between the mean center, and *X* and *Y*.

Length of *XY* axis:

(11) $$\sigma_{x}=\sqrt{2}\sqrt{\frac{\sum_{i=1}^{n}{\left(\tilde{x_{i}}cos\theta -\tilde{y_{i}}sin\theta \right)}^{2}}{n}},$$

(12) $$\sigma_{y}=\sqrt{2}\sqrt{\frac{\sum_{i=1}^{n}{\left(\tilde{x_{i}}cos\theta +\tilde{y_{i}}sin\theta \right)}^{2}}{n}},\;$$

$\sigma_{x}$ and $\sigma_{y}$ represent the standard deviation along the *x* and *y* axes, respectively.

### 3.6. Coupling Coordination Degree Model

The coupling coordination degree model can be used to evaluate the coupling and coordination state of water ecology and the water environment, and to identify the short board factors. On the basis of data standardization, CV-TOPSIS and the coupling coordination degree model are used for calculations. The specific steps include:

1. Data standardization and weight calculation. The range method is used to standardize the data, and the coefficient of variation method is used to calculate the weight of each index (Table 3).
2. The CV-TOPSIS method is used to calculate the comprehensive evaluation value.

Calculation specification matrix:

(13) $$Z_{ij}=\frac{y_{ij}}{\sqrt{\sum_{i=1}^{n}y_{ij}^{2}}},$$

Calculate the weighted gauge matrix:

(14) $$U_{ij}=w_{i}\cdot z_{ij},$$

Calculate the distance between each scheme and the ideal solution as the evaluation value:

(15) $$C_{i}^{*}=\frac{\sqrt{\sum_{j=1}^{n}{\left(U_{ij}-U_{j}^{o}\right)}^{2}}}{\sqrt{\sum_{j=1}^{n}{\left(U_{ij}-U_{j}^{o}\right)}^{2}}+\sqrt{\sum_{j=1}^{n}{\left(U_{ij}-U_{j}^{*}\right)}^{2}}}$$

where the ideal solution is: $\;\;U_{j}^{*}=\max\left(U_{1},U_{2}\ldots ,U_{j}\right)$; the negative ideal solution is: $\;U_{j}^{0}=\min\left(U_{1},U_{2}\ldots ,U_{j}\right)$.

3. Calculation of coupling coordination degree.

(16) Bi={ENi*×ECi*[(ENi*+ECi*)/2]2}12,

where $EN_{i}^{*}$, $EC_{i}^{*}$ are the evaluation value of the water environment and water ecosystem calculated by the weighted TOPSIS method.

Calculate coupling co scheduling:

(17) $$D^{i}=\sqrt{B_{i}\times \left(\alpha EN_{i}^{*}+\beta EC_{i}^{*}\right)},$$

where “*α*” and “*β*” represent the importance of the water environment and water ecology, which are equally important in WEE protection. Therefore, *α* and *β* are defined as 0.5 and 0.5, respectively, in this study. The classification standard of reference [62] and the classification of coupling coordination degree of the WEE are shown in Table 5.

### 3.7. Data Sources

The data were collected from 2007 to 2019. The data of indicators of the water environment dimension are mainly obtained from China’s urban statistical yearbook, provincial and municipal statistical yearbooks, and a small part of environmental data are obtained through investigation and interpolation. The data of indicators of water ecological dimension are mainly calculated by referring to The Manual on the Production and Emission Coefficient of Industrial Pollution Sources in the First China Pollution Source Survey. The mining volume of main metal mineral resources in mineral resources of mining cities are mainly obtained through the China Mining Yearbook and the mineral resources development and utilization database of the Ministry of Natural Resources, and the heavy metal emission coefficient of the mineral resources is obtained through The Manual on the Production and Emission Coefficient of Industrial Pollution Sources in the First China Pollution Source Survey. 

## 4. Results

### 4.1. WEE Assessment

**Figure 5:**
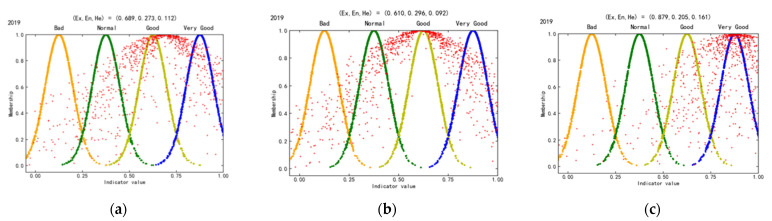
WEE cloud maps in mining cities in the YREB. (**a**) WEE index. (**b**) Water environment. (**c**) Water ecology.

Based on the cloud model theory, the cloud model parameters Ex, En, and He are calculated and determined. Figure 5 shows that the WEE index and water environment are generally suitable, and the water ecology is highly suitable in mining cities in the YREB in 2019. The He of the water ecology dimension is the largest (0.161), indicating that its uncertainty is higher than that of the water environment dimension. It can be observed that the overall level of water ecology of mining cities in the YREB is better than that of water environment, but the former takes greater fluctuation risk than the latter. Therefore, we should improve the water environment quality of most mining cities and pay attention to the water ecological safety of some key mining cities.

**Figure 6:**
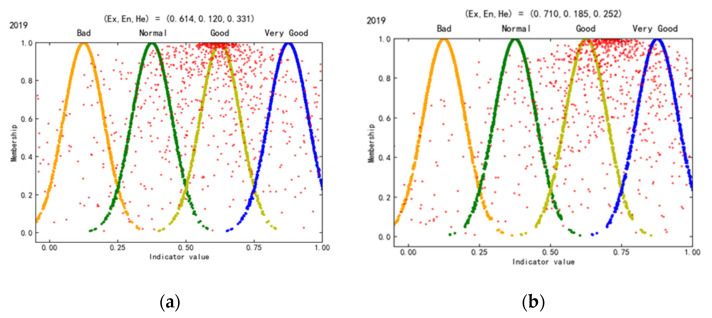
WEE cloud maps in mid-upstream and downstream mining cities in the YREB. (**a**) Mid-upstream mining cities. (**b**) Downstream mining cities.

The WEE of the mid-upstream and downstream mining cities is acceptable (Figure 6). Ex (0.71) of downstream mining cities is greater than that of mid-upstream mining cities (0.641), indicating that the WEE of the former is slightly better than that of the latter. Similarly, He (0.128) of downstream mining cities is greater than that of mid-upstream mining cities (0.122), which indicates that the WEE fluctuation risk of downstream mining cities is greater.

### 4.2. Temporal and Spatial Distribution Characteristics of the WEE

**Figure 7:**
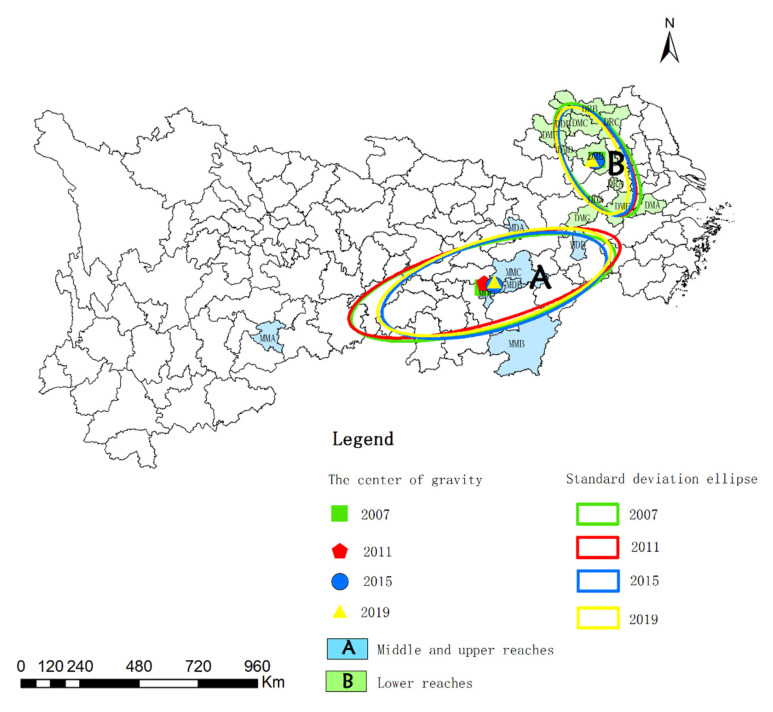
Standard deviation ellipse of the WEE of mining cities in different regions.

Table 6 and Figure 7 show the standard deviation ellipse of the WEE of mining cities in different regions. From the center of gravity transfer perspective, the center of gravity of the WEE of mid-upstream mining cities is transferred from MDC to MMC from 2007 to 2019, and the center of gravity of the WEE moved 0.04° longitudinally and 0.2° latitudinally. The center of gravity of the WEE of downstream mining cities has always been DMB, and the center of gravity of the WEE has moved 0.19° longitudinally and 0.02° latitudinally. Considering the coverage, MDC and MDB are the main areas of the WEE of mid-upstream mining cities, and DMB, DMC, and DRA are the main areas of the WEE of downstream mining cities. From 2007 to 2019, the length of the long semi axle in mid-upstream and downstream mining cities decreased from 4.98 km to 4.42 km, and from 2.35 km to 2.12 km, respectively. The length of the short semi axle in mid-upstream mining cities increased from 1.54 km to 1.57 km, and decreased from 1.10 km to 1.02 km in downstream mining cities. This indicates that the WEE of mid-upstream mining cities is shrinking in the main direction and expanding in the secondary direction, while the WEE of downstream mining cities is shrinking in the main and secondary directions. The long and short axis ratios of downstream mining cities is significantly higher than those in the mid-upstream, indicating that the WEE quality of downstream mining cities is better. The standard deviation ellipse azimuth is used to reflect the main trend direction of spatial distribution. The ellipse azimuth of mid-upstream mining cities is expanded from 75.01° to 73.32°, and the ellipse azimuth of downstream mining cities is expanded from 146.85° to 153.23°. The ellipse axis shows a clockwise rotation trend.

**Figure 8:**
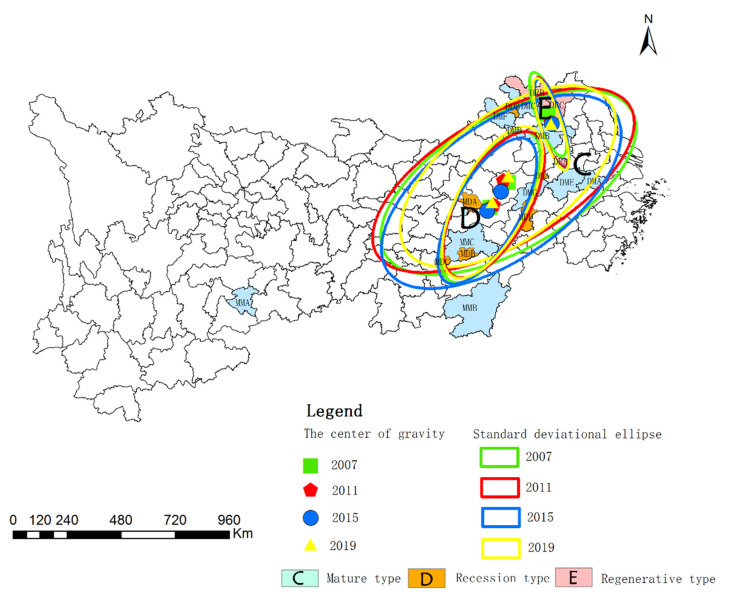
Standard deviation ellipse of the WEE of mining cities in different stages.

Table 7 and Figure 8 show the standard deviation ellipse of the WEE in mining cities at different stages. From 2007 to 2019, the center of gravity of the WEE in mature, declining, and renewable mining cities changes little, and the change of the longitude and latitude is not evident. In mature cities, the lengths of the long and short semi axles are shortened from 5.70 km to 5.03 km, and 2.67 km to 2.52 km, respectively. There is little change in the declining mining cities. In regenerative mining cities, the length of long semi axle is expanded from 1.77 km to 1.92 km, with shortening from 0.26 km to 0.22 km considering length of the short semi axle. It indicates that the WEE of mature mining cities is shrinking in the main and secondary directions, while renewable mining cities are expanding in the main direction and shrinking in the secondary trend direction. It can be seen that the long-short axis ratio of mature mining cities is greater than that of the declining and regenerative, indicating that the WEE of mature mining cities is better. The standard deviation ellipse azimuth is used to reflect the main trend direction of spatial distribution. The ellipse azimuth of mature and declining mining cities are reduced from 59.80° to 53.43°, and 28.49° to 28.43°, respectively, but that of the regenerative mining cities is expanded from 160.13° to 161.99°. The elliptical axis of mature and declining mining cities rotate counterclockwise, and the elliptical axis of regenerative mining cities shows a clockwise rotation trend.

**Figure 9:**
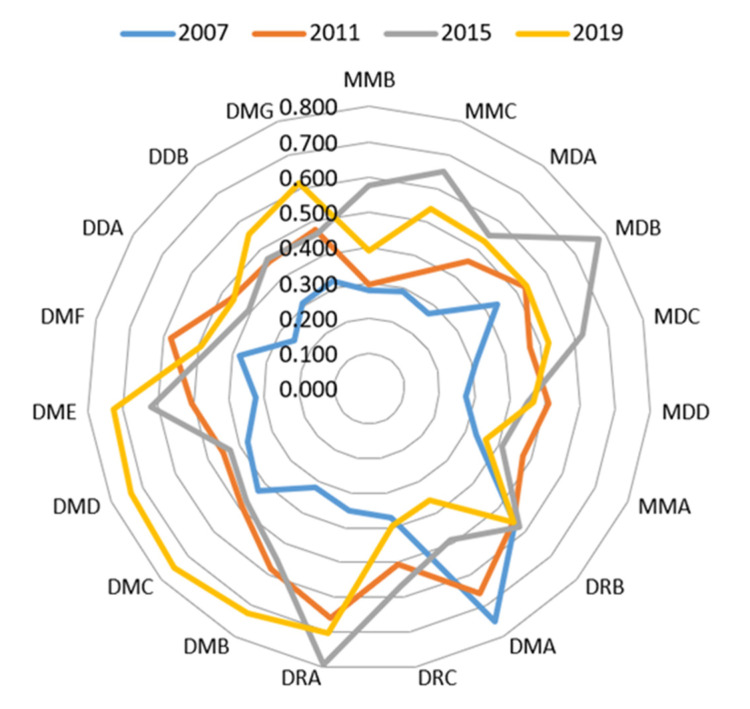
WEE index of mining cities in 2007, 2011, 2015, and 2019.

Figure 9 shows the WEE index of mining cities in 2007, 2011, 2015, and 2019. From the perspective of time change, the WEE index of many mining cities shows an upward trend, and the WEE index is the largest in 2015 or 2019. In terms of the spatial distribution, most mid-upstream mining cities had the highest WEE index in 2015, accounting for approximately 71.43%; 50% of the downstream mining cities have the highest WEE index in 2019. Among them, the mid-upstream mining cities with the highest WEE index in 2015 accounted for 62.5%. It can be seen that the WEE quality of most mining cities has been continuously optimized over the past five years. The WEE quality of downstream mining cities is better than that of mid-upstream mining cities in 2019, and improved extensively than that in 2007. The three mining cities with the largest increase in the WEE index in 2019 compared with 2007 are DME (126.38%), DMB (126.78%), and DRA (99.56%). Simultaneously, according to the development stage types of mining cities with the highest WEE index, the WEE index of mature mining cities is the highest. The above results further verify the results of the standard deviation ellipse.

### 4.3. Coupling Coordination Degree

**Figure 10:**
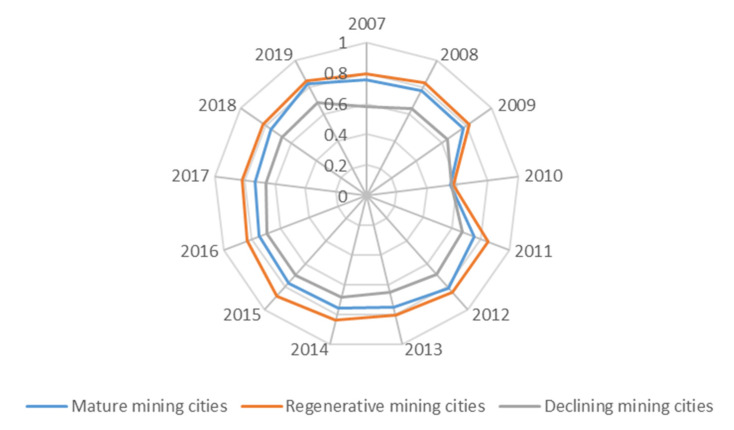
Average value of the coupling coordination degree in different types of mining cities from 2007 to 2019.

**Figure 11:**
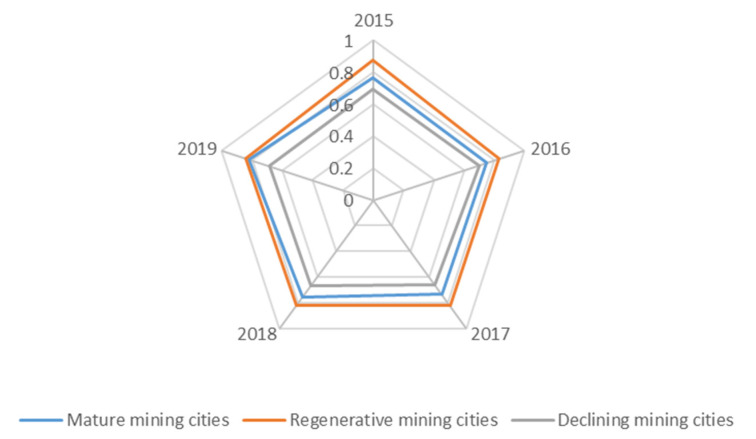
Average value of the coupling coordination degree in different types of mining cities from 2015 to 2019.

Generally speaking, the coupling coordinated development between the water environment and water ecology in mature and renewable mining cities is in a well-coordinated development state in the initial stage, and the trend of coupling coordination is basically balanced (Figure 10). From 2015 to 2019, the overall level of water environment and water ecology coupling coordination degree of renewable mining cities is higher than that of mature mining cities. The average value range of the coupling coordination degree of renewable mining cities is [0.7501, 0.9228], and the average value range of mature mining cities is [0.6644, 0.8238]. The water environment and water ecology of declining mining cities fluctuates frequently, and the average value range of the coupling coordination degree in past five years is [0.0602, 0.8758] (Figure 11).

**Figure 12:**
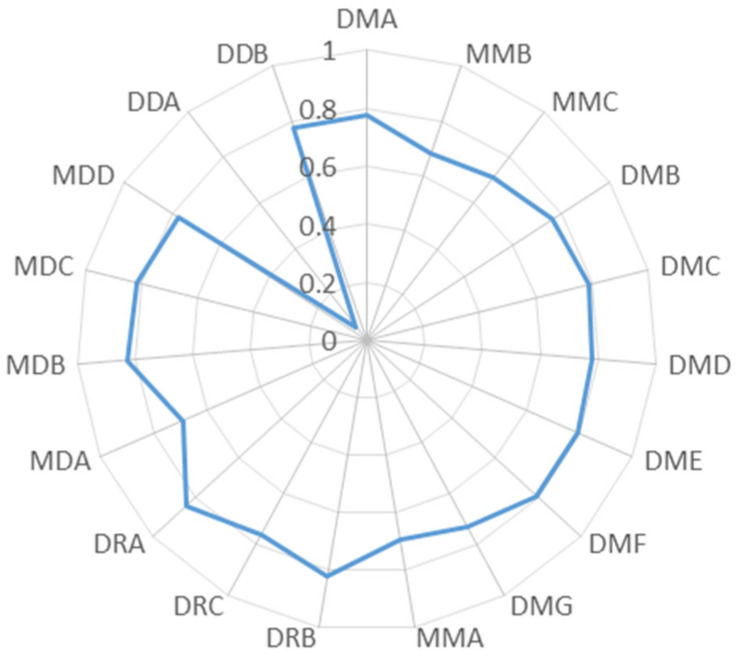
Average value of the coupling coordination degree of each mining city from 2007 to 2019.

The coupling coordination degree of mining cities is shown in Figure 12. The average coupling coordination degree of DMF reaches 0.795, the highest among the mature mining cities. In 2008, the coupling coordination degree of DMF reaches 0.8182, achieving a good coordination stage and maintaining a stable trend compared with other mature mining cities. The coupling coordination degree of MDB and MDC are higher than those in declining mining cities. However, the coupling coordination degree in DDA is lower than 0.4, which demonstrates the unbalanced of WEE. MDA, MDD, and DDB are in moderate coordination. The coupling coordination degree of DRA is higher than DRB and DRC. The average coupling coordination degree of DRB is higher than 0.8, which is in a high coordination. The coupling coordination degree between DRA and DRB increased slightly, and the average coupling coordination degree of DRC reaches 0.7649, which is in a moderate coordination.

Most mining cities still belong to a lagging water environment. Table 8 shows that 17 mining cities belong to a water environment lag type, accounting for 89.5%, and 2 belong to a water ecology lag type, accounting for 10.5%.

## 5. Discussion

The WEE of mining cities in the YREB is generally good, but there is still room for improvement. Efforts should be made to improve the construction of sewage pipelines in mining cities for optimizing the quality of the water environment. According to the weight data, the indicator of length of sewage pipeline accounts for the highest proportion in the index system of the WEE, which is in line with China’s saying that “black odor is in the water, the root is on the shore, the key is the outlet, and the core is the pipe network.” Optimizing the construction of sewage pipelines in mining cities under the background of China’s new infrastructure is one of the starting points for improving the quality of the WEE. Conversely, mining cities that fall behind in the construction of water ecological civilization should be the point of focused. This also indicates the necessity of analyzing spatial distribution characteristics for locating these mining cities.

Compared with 2007, WEE protection in most mining cities has achieved remarkable results. This agrees with the findings of Yang (2019) that the ecological quality in most regions of the Yangtze River Basin has remained the same or increased [63], and of Zhou (2021) and Deng (2021) that the emissions of water pollutants COD and NH_3_-N in the YREB have been decreasing year by year [64,65]. In November 2012, the construction of ecological civilization was evidently proposed in the 18th National Congress of the Communist Party of China, and then The Water Pollution Prevention and Control Action Plan, The Ecological Environment Protection Plan of YREB, The Outline of The Regional Integration Development Plan of The Yangtze River Delta, The Yangtze River Water Protection Law, and other documents were intensively issued, which indicates the direction for WEE protection of mining cities in the YREB. This also reflects the advantage of the party and government leaders of the Communist Party of China in “concentrating on major events” [66], and “joint protection and no large-scale development” of the YREB has been implemented. The WEE quality of mature mining cities in the downstream is better than that of other types of mining cities in 2019, and the maturity has made greater progress than that in 2007, which is consistent with the fact that nonferrous and ferrous metals in the mid-upstream of the YREB play an important role in local economic and social development and a positive role in China’s resource protection. In addition, mature mining cities in the downstream have achieved remarkable results during the construction of sewage pipelines.

The coordinated development of water environment and water ecology in regenerative mining cities is better than that in mature and declining mining cities. Transformation is the only way for China’s mining cities for achieving sustainable development [67]. Both mature and declining mining cities are facing transformation, and regenerative mining cities have basically completed economic transformation. Developing mining cities into tourist destinations has been adopted by many cities [68], such as regenerative mining cities Lijiang City and Shangri-La County. The transformation of mining cities is often planned after they enter a recession [69], i.e., most mature and declining mining cities plan the economic transformation late, and mining development remains their focus. Therefore, the focus should be on coordinated development of water environment and water ecology in regenerative mining cities.

Most mining cities still belong to the lagging type of water environment. Heavy metal pollution has been well treated, and the threat to water ecological security caused by heavy metal pollution is low. Huang (2020) also mentioned that the pollution of Cd, Pb, As, Cu, and Zn in the YREB have been reduced [70], and Yan (2021) noted that heavy metal pollution in surface water in the YREB has decreased in recent years [71]. These studies all confirm our conclusion. Heavy metals are toxic, persistent, and bio-aggregative. Aquatic ecosystems have attracted much attention worldwide because of their heavy metal pollution [72,73]. In The 12th Five Year Plan for Comprehensive Prevention and Control of Heavy Metal Pollution, China defined 138 key protection areas and 4452 key prevention and control enterprises to tackle heavy metal pollution by classification and zoning. The comprehensive assessment results of the implementation of the above plan show that in 2015, the total discharge of major heavy metal pollutants in China decreased by 27.6% compared with 2007, and less than 3 heavy metal-related environmental emergencies occurred annually from 2012 to 2015 [74].

## 6. Conclusions and Recommendations

Based on the above research, the following conclusions were drawn: (1) the WEE of mining cities in the YREB is generally good, but efforts should be made to improve the construction of sewage pipelines in mining cities, and attention should be paid to mining cities that lag in constructing ecological water civilization. (2) Recently, remarkable results have been achieved in WEE protection of most mining cities. The WEE quality of mature mining cities in the downstream is better than that of other types of mining cities in 2019, which is greater than that in 2007. (3) The coordinated development of water environment and water ecology in regenerative mining cities is better than that in the mature and declining cities. (4) Most mining cities still belong to the lagging type of water environment. Heavy metal pollution has been well treated, and the threat to water ecological security caused by heavy metal pollution is low.

To solve the WEE problems existing in the development of mineral resources, “joint protection and no large-scale development” should be followed, and the relationship between development and the bottom line should be coordinated. The specific recommendations are as follows.

(1) Improve the sewage pipe network system of mining cities. The improvement of urban and rural drainage pipe network construction should be accelerated to establish 100% coverage and collection of sewage pipe network in city proper as soon as possible. WEE protection awareness of rural residents should be improved by public service advertising. Both the treatment of rural domestic sewage and the toilet revolution and the treatment of livestock and poultry manure should be promoted scientifically and reasonably.
(2) Promote the treatment of the WEE in the mid-upstream mining cities further. The mid-upstream in the YREB has the most mines and is rich in strategic metal minerals. While developing strategic metal minerals and ensuring the safety of China’s resources, a strict environmental protection system should be implemented. For 306 key mining agglomeration areas in the YREB, the discharge of wastewater and solid waste from various activities should be strictly controlled for ensuring that the discharge of various pollutants meets the discharge standards, and curbing the water environment pollution in mining areas and parks. Ecological compensation for the ecological functional areas in the mid-upstream should be increased to alleviate the economic and social pressure of WEE treatment.
(3) The transformational development of mature and declining mining cities should be arranged in advance. The efficiency of resource development and utilization and industrial technology should be improved in mature mining cities, and the resource industrial chain should be extended for cultivating resource deep-processing industrial clusters and build leading enterprises. Meanwhile, the adjustment and upgrading of resource processing industrial structure should be accelerated, and the formation of corresponding pillar alternative industries should be promoted. In declining mining cities, the problem of urban internal dual structure and left over by history, such as accelerating the comprehensive treatment of hidden dangers of geological disasters, should be solved. With China’s policy support, the development of alternative industries should be vigorously supported to constantly enhance their sustainable development capacity.

There are limitations to the analysis performed in this study. In establishing the water ecological indicator system, the quantities of fish, shrimp, and microorganisms are more informative indicators of the water quality. However, this data is rarely available owing to the degree of work involved in determining the numbers of these aquatic organisms. Therefore, mercury, cadmium, lead, and arsenic emissions were used as the indicators of water quality from a different perspective. In future studies, we will focus on the biological accounting of aquatic organisms, as well as explore the means to improve the water environment while assessing the impact of various policies on this specific environment.

## Figures and Tables

**Table 1:** List of study areas.

Scope	City	Stage	Code
Mid-upstream	Anshun	Mature	MMA
	Ganzhou	Mature	MMB
	Yichun	Mature	MMC
	Huangshi	Declining	MDA
	Xinyu	Declining	MDB
	Pingxiang	Declining	MDC
	Jingdezhen	Declining	MDD
Downstream	Huzhou	Mature	DMA
	Chuzhou	Mature	DMB
	Suzhou	Mature	DMC
	Huainan	Mature	DMD
	Yicheng	Mature	DME
	Haozhou	Mature	DMF
	Chizhou	Mature	DMG
	Tongling	Declining	DDA
	Huaibei	Declining	DDB
	Maanshan	Regenerative	DRA
	Xuzhou	Regenerative	DRB
	Suqian	Regenerative	DRC

**Table 2:** Identification of water pollution impact factors in provinces (cities), YREB.

Province	Surface Water Pollution	Groundwater Pollution	Water Source and Reservoir Pollution
Guizhou	▲☆◇	▲★◆	▲★◆
Yunnan	▲★◇	▲★◇	-
Sichuan	△☆◇	▲★◇	-
Chongqing	△☆◇	▲★◆	▲☆◇
Hunan	▲☆◇	▲★◆	▲★◇
Hubei	▲☆◇	▲★◆	▲★◇
Jiangxi	-	-	-
Anhui	▲☆◇	▲★◆	▲★◇
Zhejiang	△☆◇	△☆◆	△☆◇
Jiangsu	▲☆◇	▲★◆	▲★◇
Shanghai	▲☆◇	△☆◆	△☆◇

(Degree of impact: ▲ remarkable, △ slight; aging of impact: ★ long term, ☆ short term; category of impact: ◆ irreversible, ◇ reversible).

**Table 3:** Evaluation index system of the WEE system.

Dimensionality	Indicator	Attribute	Weight
Water environment	Discharge of industrial wastewater (10 kt)	-	0.120789
	Discharge of industrial sulfur dioxide (t)	-	0.107262
	Length of sewage pipelines (km)	+	0.226373
	Discharge of industrial (tobacco) dust (t)	-	0.108185
	Discharge of industrial COD (t)	-	0.09297
	Discharge of industrial ammonia nitrogen (t)	-	0.068896
Water ecology	Discharge of mercury (mg/t)	-	0.067365
	Discharge of cadmium (g/t)	-	0.067581
	Discharge of plumbum (g/t)	-	0.068432
	Discharge of arsenic (g/t)	-	0.072146

**Table 4:** Standard for classification of WEE systems.

Level	Interval Partition	Standard Cloud Parameter	Description
1	[0, 0.25)	(0.125, 0.0736, 0.001)	Bad
2	[0.25, 0.5)	(0.375, 00.0736, 0.001)	Normal
3	[0.5, 0.75)	(0.625, 0.0736, 0.001)	Good
4	[0.75, 1)	(0.875, 0.0736, 0.001)	Very Good

**Table 5:** Classification of coupling coordination degree of the WEE.

Range	Grade
(0, 0.4]	Unbalanced
(0.4, 0.5]	Basic balanced
(0.5, 0.8]	Moderate coordination
(0.8, 1]	High coordination

**Table 6:** Standard deviation ellipse parameters of the WEE of mining cities in different regions.

Region	Year	2007	2011	2015	2019
Mid-upstream	Key City	MDC	MDC	MMC	MMC
	Center	113.77° E, 27.75° N	113.81° E, 27.91° N	114.17° E, 27.83° N	114.17° E, 27.95° N
	Elliptical area/km^2^	24.08	23.73	21.46	21.74
	Length of long half shaft/km	4.98	5.14	4.24	4.42
	Length of short half shaft/km	1.54	1.47	1.61	1.57
	Long and short axis ratio	0.31	0.29	0.38	0.36
	Rotation	75.01	74	74.06	73.32
Downstream	Key City	DMB	DMB	DMB	DMB
	Center	117.94° E, 32.41° N	117.88° E, 32.36° N	117.89° E, 32.37° N	117.75° E, 32.39° N
	Elliptical area/km^2^	8.14	7.59	7.28	6.82
	Length of long half shaft/km	2.35	2.21	2.17	2.12
	Length of short half shaft/km	1.1	1.09	1.07	1.02
	Long and short axis ratio	0.47	0.49	0.49	0.48
	Rotation	146.85	149.06	151.83	153.23

**Table 7:** Standard deviation ellipse parameters of the WEE of mining cities in different stages of the YREB.

Stage	Year	2007	2011	2015	2019
Mature	Center	116.53° E, 30.70° N	116.33° E, 30.79° N	116.24° E, 30.33° N	116.52° E, 30.94° N
	Elliptical area/km^2^	47.79	47.27	46.79	39.76
	Length of long half shaft/km	5.7	5.86	5.54	5.03
	Length of short half shaft/km	2.67	2.57	2.69	2.52
	Long and short axis ratio	0.47	0.44	0.49	0.5
	Rotation	59.8	59.93	54.97	53.43
Declining	Center	115.81° E, 29.72° N	115.94° E, 29.81° N	115.68° E, 29.56° N	115.88° E, 29.90° N
	Elliptical area/km^2^	14.11	14.38	13.34	14.59
	Length of long half shaft/km	3.36	3.26	3.27	3.36
	Length of short half shaft/km	1.34	1.4	1.3	1.38
	Long and short axis ratio	0.4	0.43	0.4	0.41
	Rotation	28.49	29.78	29.77	28.43
Regenerative	Center	118.11° E, 33.42° N	118.23° E, 33.11° N	118.26° E, 33.03° N	118.22° E, 33.02° N
	Elliptical area/km^2^	2.58	2.64	2.62	2.56
	Length of long half shaft/km	1.77	1.86	1.87	1.92
	Length of short half shaft/km	0.46	0.45	0.45	0.42
	Long and short axis ratio	0.26	0.24	0.24	0.22
	Rotation	160.13	162.53	163.05	161.99

**Table 8:** Lag types of water environment and water ecology coordinated development of mining cities in the YREB in 2019.

Lag Type	Quantity	Mining Cities
Water environment	17	MMA, MMB, MMC, MDB, MDC, MDD, DMA, DMB, DMC, DMD, DME, DMF, DMG, DDB, DRA, DRB, DRC
Water ecology	2	MDA, DDA

## Data Availability

The data presented in this study are available in China’s urban statistical yearbook, provincial and municipal statistical yearbooks, The Manual on the Production and Emission Coefficient of Industrial Pollution Sources in the First China Pollution Source Survey.

## References

[1] Xi Jinping, Delivered at the 19th National Congress of the Communist Party of China. http://cpc.people.com.cn/n1/2017/1028/c64094-29613660.html.

[2] Varol M. (2020). Use of water quality index and multivariate statistical methods for the evaluation of water quality of a stream affected by multiple stressors: A case study. J. Environ. Pollut..

[3] Ministry of Environmental Protection, National Development and Reform Commission, Ministry of Water Resources, Ministry of Ecological Environment of the People’s Republic of China (2017). Ecological Environment Protection Planning of the Yangtze River Economic Belt. https://www.zhb.gov.cn/gkml/hbb/qt/201707/t20170718_418074.htm.

[4] Bridge G. (2004). Contested Terrain: Mining and the Environment. J. Annu. Rev. Environ. Resour..

[5] Zhang J., Liu C.L. (2002). Riverine composition and estuarine geochemistry of particulate metals in China—Weathering features, anthropogenic impact and chemical fluxes. J. Estuar. Coast. Shelf Sci..

[6] Birch G.F., Chang C.H., Lee J.H., Churchill L.J. (2013). The use of vintage surficial sediment data and sedimentary cores to determine past and future trends in estuarine metal contamination (Sydney estuary, Australia). J. Sci. Total Environ..

[7] Zou S.R., Du S.X., Song M., Li M.X. (2021). How Polluting Industries React to Ambient Water Quality: Seven River Basins in China. J. Water.

[8] Dos Santos E.S., Lopes P.P.P., da Silva Pereira H.H., de Oliveira Nascimento O., Rennie C.D., O’Reilly L.D.S.L., da Cunha A.C. (2018). The impact of channel capture on estuarine hydro-morphodynamics and water quality in the Amazon delta. J. Sci. Total Environ..

[9] Avila R., Horn B., Moriarty E., Hodson R., Moltchanova E. (2018). Evaluating statistical model performance in water quality prediction. J. Environ. Manag..

[10] Li M., Zhang J., Luo H., Liang N., Yu Y., Sun Y. (2011). Correlation analysis between China’s chemical oxygen demand emission reduction and changes in water environment quality during the Eleventh Five Year Plan. J. Ecol. Environ..

[11] Wei X., Wang J., Wu S., Xin X., Wang Z., Liu W. (2019). Comprehensive evaluation model for water environment carrying capacity based on VPOSRM framework: A case study in Wuhan, China. J. Sustain. Cities Soc..

[12] Zhang Y., Yue Q., Wang T., Zhu Y., Li Y. (2021). Evaluation and early warning of water environment carrying capacity in Liaoning province based on control unit: A case study in Zhaosutai River Tieling City control unit. J. Ecol. Indic..

[13] Duan T.T., Feng J.S., Zhou Y.Q., Chang X., Li Y.X. (2021). Systematic evaluation of management measure effects on the water environment based on the DPSIR-Tapio decoupling model: A case study in the Chaohu Lake watershed, China. J. Sci. Total Environ..

[14] Simeonov V., Stratis J.A., Samara C., Zachariadis G., Voutsa D., Anthemidis A., Sofoniou M., Kouimtzis T. (2003). Assessment of the surface water quality in Northern Greece. J. Water Res..

[15] Wan R., Meng F., Su E., Fu W., Wang Q. (2018). Development of a classification scheme for evaluating water quality in marine environment receiving treated municipal effluent by an integrated biomarker approach in *Meretrix meretrix*. J. Ecol. Environ..

[16] Ji X.L., Wang X., Yang G.P. (2020). A water quality assessment model for Suya Lake Reservoir. J. Water Supply Dec..

[17] Xing L., Xue M., Hu M. (2019). Dynamic simulation and assessment of the coupling coordination degree of the economy–resource–environment system: Case of Wuhan City in China. J. Environ. Manag..

[18] Yang C., Zeng W., Yang X. (2020). Coupling coordination evaluation and sustainable development pattern of geo-ecological environment and urbanization in Chongqing municipality, China. J. Sustain. Cities Soc..

[19] Kavka P. (2021). Spatial Delimitation of Small Headwater Catchments and Their Classification in Terms of Runoff Risks. J. Water.

[20] Zuo Z., Guo H., Cheng J., Li Y. (2021). How to achieve new progress in ecological civilization construction?—Based on cloud model and coupling coordination degree model. J. Ecol. Indic..

[21] Liu N., Liu C., Xia Y., Da B. (2018). Examining the coordination between urbanization and eco-environment using coupling and spatial analyses: A case study in China. J. Ecol. Indic..

[22] Li W., Wang Y., Xie S., Cheng X. (2021). Coupling coordination analysis and spatiotemporal heterogeneity between urbanization and ecosystem health in Chongqing municipality, China. J. Sci. Total Environ..

[23] Wang R., Cheng J., Zhu Y., Xiong W. (2016). Research on diversity of mineral resources carrying capacity in Chinese mining cities. J. Resour. Policy.

[24] Zeng L., Wang B., Fan L., Wu J. (2016). Analyzing sustainability of Chinese mining cities using an association rule mining approach. J. Resour. Policy.

[25] Tai X., Xiao W., Tang Y. (2020). A quantitative assessment of vulnerability using social-economic-natural compound ecosystem framework in coal mining cities. J. Clean. Prod..

[26] Zhou M., Li X., Zhang M., Liu B., Zhang Y., Gao Y., Ullah H., Peng L., He A., Yu H. (2020). Water quality in worldwide coal mining city: A scenario in water chemistry and health risks exploration. J. Geochem. Explor..

[27] Xi X., Wang S., Yao L., Zhang Y., Niu R., Zhou Y. (2021). Evaluation on geological environment carrying capacity of mining city—A case study in Huangshi City, Hubei Province, China. Int. J. Appl. Earth Obs. Geoinf..

[28] Li X., Li Y., Lu Z. (2016). Study on distribution characteristics of heavy metal pollution in mineral resources development—Taking a copper mine area in Yalong river flow area as an example. J. Miner. Prot. Util..

[29] Zhang C., Qiao Q., Piper J.D., Huang B. (2011). Assessment of heavy metal pollution from a Fe-smelting plant in urban river sediments using environmental magnetic and geochemical methods. J. Environ. Pollut..

[30] Dong C., Zhang W., Ma H., Feng H., Lu H., Dong Y., Yu L. (2014). A magnetic record of heavy metal pollution in the Yangtze River subaqueous delta. J. Sci. Total Environ..

[31] Chen X., Jiang C., Zheng L., Zhang L., Fu X., Chen S., Chen Y., Hu J. (2021). Evaluating the genesis and dominant processes of groundwater salinization by using hydrochemistry and multiple isotopes in a mining city. J. Environ. Pollut..

[32] Cui D., Chen X., Xue Y., Li R., Zeng W. (2019). An integrated approach to investigate the relationship of coupling coordination between social economy and water environment on urban scale—A case study of Kunming. J. Environ. Manag..

[33] Tian Y., Zhou D., Jiang G. (2020). Conflict or Coordination? Multiscale assessment of the spatio-temporal coupling relationship between urbanization and ecosystem services: The case of the Jingjinji Region, China. J. Ecol. Indic..

[34] Li J., Sun W., Li M., Meng L. (2021). Coupling Coordination Degree of Production, Living and Ecological Spaces and its Influencing Factors in the Yellow River Basin. J. Clean. Prod..

[35] Ariken M., Zhang F., Chan N., Kung H. (2020). Corrigendum to “Coupling coordination analysis and spatio-temporal heterogeneity between urbanization and eco-environment along the Silk Road Economic Belt in China”. J. Ecol. Indic..

[36] Yang Y., Bao W., Liu Y. (2020). Coupling coordination analysis of rural production-living-ecological space in the Beijing-Tianjin-Hebei region. J. Ecol. Indic..

[37] Dong F., Li W. (2021). Research on the coupling coordination degree of “upstream-midstream-downstream” of China’s wind power industry chain. J. Clean. Prod..

[38] Yeh C.H. (2003). The selection of multiattribute decision making methods for scholarship student selection. J. Int. J. Sel. Assess..

[39] Chen P. (2019). Effects of normalization on the entropy-based topsis method. J. Expert Syst. Appl..

[40] Sun W., Li D., Liu P. (2018). A decision-making method for sponge city design based on grey correlation degree and topsis method. J. Interdiscip. Math..

[41] Sałabun W., Ziemba P., Wątróbski J. (2016). The rank reversals paradox in management decisions: The comparison of the ahp and comet methods. Proceedings of the International Conference on Intelligent Decision Technologies.

[42] Dezert J., Tchamova A., Han D., Tacnet J.M. (2020). The SPOTIS rank reversal free method for multi-criteria decision-making support. Proceedings of the IEEE 23rd International Conference on Information Fusion (FUSION).

[43] Leng L., Mao X., Jia H., Xu T., Chen A.S., Yin D., Fu G. (2020). Performance assessment of coupled green-grey-blue systems for Sponge City construction. J. Sci. Total Environ..

[44] Zeng J., Lin G., Huang G. (2021). Evaluation of the cost-effectiveness of Green Infrastructure in climate change scenarios using TOPSIS. J. Urban For. Urban Green..

[45] Long R., Li H., Wu M., Li W. (2021). Dynamic evaluation of the green development level of China’s coal-resource-based cities using the TOPSIS method. J. Resour. Policy.

[46] Lin S.S., Shen S.L., Zhou A., Xu Y.S. (2020). Approach based on TOPSIS and Monte Carlo simulation methods to evaluate lake eutrophication levels. J. Water Res..

[47] Lin S.S., Shen S.L., Zhang N., Zhou A. (2021). Method for lake eutrophication levels evaluation: TOPSIS-MCS. J. MethodsX.

[48] Yang T., Zhang Q., Wan X., Li X., Wang Y., Wang W. (2020). Comprehensive ecological risk assessment for semi-arid basin based on conceptual model of risk response and improved TOPSIS model-a case study of Wei River Basin, China. J. Sci. Total Environ..

[49] Yang Y. (2021). Expansion and evolution of a typical resource-based mining city in transition using the Google Earth engine: A case study of Datong, China. J. Remote Sens..

[50] Camp B.L.M., Nel V., Mphambukeli T. (2017). A thriving coal mining city in crisis? The governance and spatial planning challenges at Witbank, South Africa. Land Use Policy.

[51] Peng K., Luo C., Lou L., Li X., Shen Z. (2008). Bioaccumulation of heavy metals by the aquatic plants *Potamogeton pectinatus* L. and *Potamogeton malaianus* Miq. and their potential use for contamination indicators and in wastewater treatment. Sci. Total Environ..

[52] Salehpour Jam A., Mosaffaie J., Sarfaraz F., Shadfar S., Akhtari R. (2021). GIS-based landslide susceptibility mapping using hybrid MCDM models. J. Nat. Hazards.

[53] Li D., Cheung D., Shi X., Ng V. (1998). Uncertainty reasoning based on cloud models in controllers. J. Comput. Math. Appl..

[54] Wang J., Zhai T., Lin Y., Kong X., He T. (2019). Spatial imbalance and changes in supply and demand of ecosystem services in China. J. Sci. Total Environ..

[55] Liu D., Wang D., Wu J., Wang Y., Wang L., Zou X., Chen Y., Chen X. (2014). A risk assessment method based on RBF artificial neural network-cloud model for urban water hazard. J. Intell. Fuzzy Syst..

[56] Li D., Liu C.Y., Liu L.Y. (2004). Study on the universality of the normal cloud model. J. Eng. Sci..

[57] Liu Y., Chen H.Y., Wang X.J. (2021). Research on green renovations of existing public buildings based on a cloud model. J. Build. Eng..

[58] Min C., Wen G., Li B., Zhao X. (2018). Comprehensive evaluation of offshore oilfield development plans based on grey clustering analysis with cloud model. J. Math. Probl. Eng..

[59] Hou M., Deng Y., Yao S. (2021). Spatial Agglomeration Pattern and Driving Factors of Grain Production in China since the Reform and Opening Up. J. Land.

[60] Tao F., Hu Y., Tang G., Zhou T. (2021). Long-term evolution of the suhi footprint and urban expansion based on a temperature attenuation curve in the Yangtze river delta urban agglomeration. J. Sustain..

[61] Ren S., Song C., Ye S., Cheng C., Gao P. (2022). The spatiotemporal variation in heavy metals in China’s farmland soil over the past 20 years: A meta-analysis. J. Sci. Total Environ..

[62] Xu S., He W., Shen J., Degefu D.M., Yuan L., Kong Y. (2019). Coupling and Coordination Degrees of the Core Water-Energy-Food Nexus in China. Int. J. Environ. Res..

[63] Yang X., Meng F., Fu P., Zhang Y., Liu Y. (2021). Spatiotemporal change and driving factors of the Eco-Environment quality in the Yangtze River Basin from 2001 to 2019. J. Ecol. Indic..

[64] Zhou K., Wu J., Liu H. (2021). Spatiotemporal variations and determinants of water pollutant discharge in the Yangtze River Economic Belt, China: A spatial econometric analysis. J. Environ. Pollut..

[65] Deng C., Li H., Peng D., Liu L., Zhu Q., Li C. (2021). Modelling the coupling evolution of the water environment and social economic system using PSO-SVM in the Yangtze River Economic Belt, China. J. Ecol. Indic..

[66] Wang R., Jia T., Qi R., Cheng J., Zhang K., Wang E., Wang X. (2021). Differentiated impact of politics- and science-oriented education on pro-environmental behavior: A case study of Chinese university students. J. Sustain..

[67] Jiao W., Zhang X., Li C., Guo J. (2021). Sustainable transition of mining cities in China: Literature review and policy analysis. J. Resour. Policy.

[68] Armis R., Kanegae H. (2020). The attractiveness of a post-mining city as a tourist destination from the perspective of visitors: A study of Sawahlunto old coal mining town in Indonesia. J. Asia-Pac. J. Reg. Sci..

[69] He S.Y., Lee J., Zhou T., Wu D. (2017). Shrinking cities and resource-based economy: The economic restructuring in China’s mining cities. J. Cities.

[70] Huang Z., Liu C., Zhao X., Dong J., Zheng B. (2020). Risk assessment of heavy metals in the surface sediment at the drinking water source of the Xiangjiang River in South China. J. Environ. Sci. Eur..

[71] Yan J., Qu Z., Li F., Li H. (2021). Heavy metals in the water environment of Yangtze River Economic Belt: Status, fuzzy environmental risk assessment and management. J. Urban Clim..

[72] Hulscher T., Mol G., Lüers F. (1992). Release of metals from polluted sediments in a shallow lake: Quantifying resuspension. J. Hydrobiol..

[73] Thomas K.V., Bijlsma L., Castiglioni S., Covaci A., Emke E., Grabic R., Hernández F., Karolak S., Kasprzyk-Hordern B., Lindberg R.H. (2018). Heavy metal pollution in reservoirs in the hilly area of southern China: Distribution, source apportionment and health risk assessment. J. Sci. Total Environ..

[74] Former Ministry of Environmental Protection of China (2016). 12th Five Year Plan for Comprehensive Prevention and Control of Heavy Metal Pollution. http://www.gov.cn/xinwen/2016-11/30/content_5140517.htm.

